# Multi-template matching: a versatile tool for object-localization in microscopy images

**DOI:** 10.1186/s12859-020-3363-7

**Published:** 2020-02-05

**Authors:** Laurent S. V. Thomas, Jochen Gehrig

**Affiliations:** 1 Acquifer is a division of Ditabis, Digital Biomedical Imaging Systems AG, Pforzheim, Germany; 2 Centre of Paediatrics and Adolescent Medicine, University Hospital Heidelberg, Heidelberg, Germany

**Keywords:** Fiji, KNIME, OpenCV, Template matching, Object-recognition, Object-localization, Pattern recognition, Classification, Zebrafish, Medaka

## Abstract

**Background:**

The localization of objects of interest is a key initial step in most image analysis workflows. For biomedical image data, classical image-segmentation methods like thresholding or edge detection are typically used. While those methods perform well for labelled objects, they are reaching a limit when samples are poorly contrasted with the background, or when only parts of larger structures should be detected. Furthermore, the development of such pipelines requires substantial engineering of analysis workflows and often results in case-specific solutions. Therefore, we propose a new straightforward and generic approach for object-localization by template matching that utilizes multiple template images to improve the detection capacity.

**Results:**

We provide a new implementation of template matching that offers higher detection capacity than single template approach, by enabling the detection of multiple template images. To provide an easy-to-use method for the automatic localization of objects of interest in microscopy images, we implemented multi-template matching as a Fiji plugin, a KNIME workflow and a python package. We demonstrate its application for the localization of entire, partial and multiple biological objects in zebrafish and medaka high-content screening datasets. The Fiji plugin can be installed by activating the Multi-Template-Matching and IJ-OpenCV update sites. The KNIME workflow is available on nodepit and KNIME Hub. Source codes and documentations are available on GitHub (https://github.com/multi-template-matching).

**Conclusion:**

The novel multi-template matching is a simple yet powerful object-localization algorithm, that requires no data-pre-processing or annotation. Our implementation can be used out-of-the-box by non-expert users for any type of 2D-image. It is compatible with a large variety of applications including, for instance, analysis of large-scale datasets originating from automated microscopy, detection and tracking of objects in time-lapse assays, or as a general image-analysis step in any custom processing pipelines. Using different templates corresponding to distinct object categories, the tool can also be used for classification of the detected regions.

## Background

In microscopy images, objects of interest usually represent only a fraction of the field of view and are randomly positioned. Typically, detection of objects in microscopy images relies on classic intensity-based segmentation techniques that perform well for the localization of fluorescent objects. However, these approaches often require the creation of complex analysis workflows and the adjustment of multiple parameters, resulting in highly application-specific solutions [1–4]. In many cases, such as whole organism imaging, such methods might not even be applicable when it comes to the identification of a particular organ or tissue that is poorly contrasted with the rest of the specimen or that is non-specifically labelled. Alternatively, methods based on the detection of edges and shapes (e.g. circular Hough transform [5]) can perform well with low contrast images, but they are limited to a given shape or are sensitive to noise. Machine learning methods offer powerful object-detection capacities [6–10]; however, setting up the software environment can be overwhelming and training the machine requires large amounts of annotated data, thus rendering it often inaccessible to most microscopy users. In contrast, template-based approaches allow the computation of the most probable positions of a single template image within a larger image with negligible manual annotation and at minimal computational cost [11]. However, using a single template, the detection capacity is limited, as the algorithm searches for a single intensity pattern which might not generalize well to objects with different perspectives or characteristics. To overcome current limitations in object-recognition, we report a new implementation of template matching with enhanced detection capacity by performing the search with multiple template images, thus improving the range of detectable patterns. The individual template detections are combined and filtered to keep the most probable detections using a custom non-maxima suppression (NMS). To address the current lack of open-source tools for generic and accessible object-detection in end-user software, we further present the design and establishment of previously unavailable multi-template-matching functionalities in Fiji [12] and KNIME [13] in a user-friendly manner.

## Implementation

Extending on OpenCV functions, we implemented multi-template matching for end-users as both a Fiji plugin and a KNIME workflow compatible with images of any bit depth. To this extent, we developed a python implementation that can be installed with its dependencies like a regular python package via pip (Multi-Template Matching). The KNIME workflow uses this python implementation via a python scripting node. The Fiji plugins rely on a collection of related Jython scripts that are automatically installed with the plugins upon activation of the *Multi-Template Matching* update site. Additionally, the *IJ-OpenCV plugins* update site [14] should be activated to install the OpenCV dependencies in Fiji. The resulting pipelines can be installed on any system running Fiji or KNIME/Python (see Additional file 13 for further details and availability of source code). A flowchart of the implementation is provided in Additional file 4: Figure S1.

**Fig. 1:**
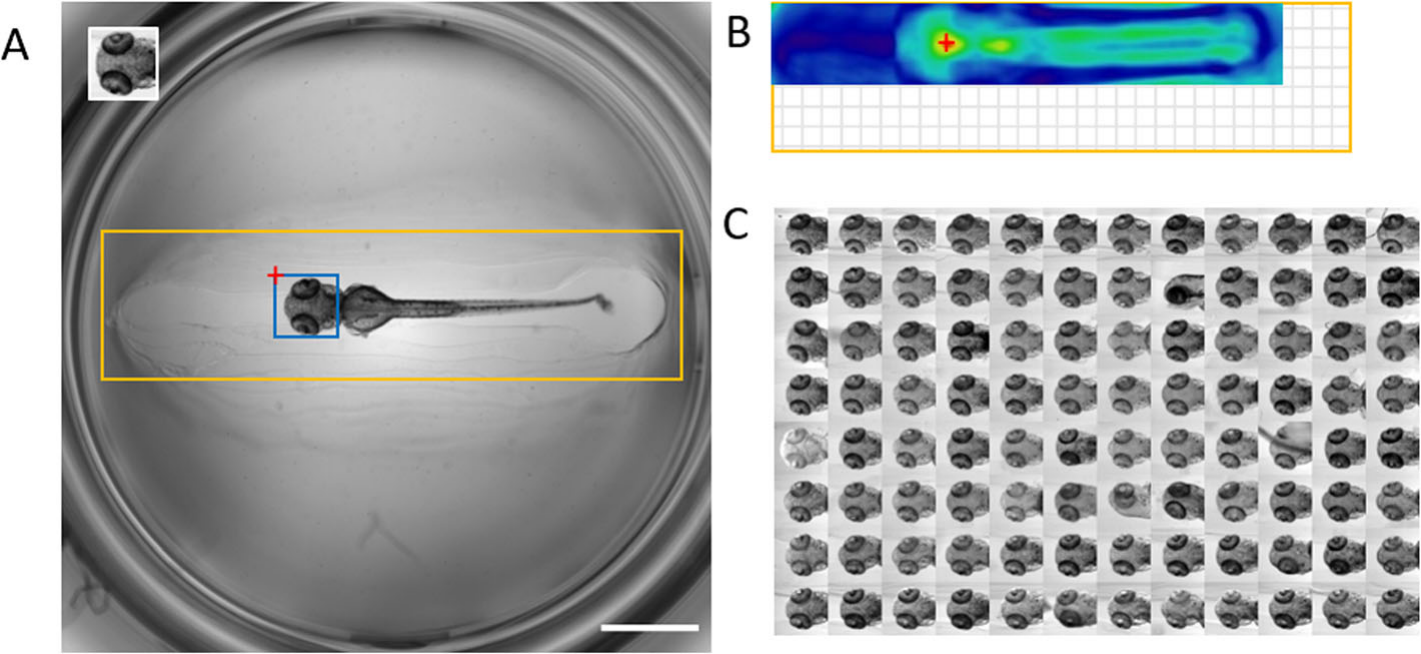
Head region detection in oriented zebrafish larvae using single template matching. **a** Searched image (2048 × 2048 pixels, scale bar: 1 mm) with template as inset (188 × 194 pixels), search region in orange (1820 × 452 pixels) and predicted location in blue. The red cross corresponds to the position of the global maximum of the correlation map (as in **b**). **b** Correlation map with global maximum (red cross) indicating the position of the bounding box in **a**. The grid area indicates the smaller size of the correlation map compared to the image in which the search is performed (see also Additional file 13). **c** Montage of detected head regions within a 96 well plate

**Fig. 2:**
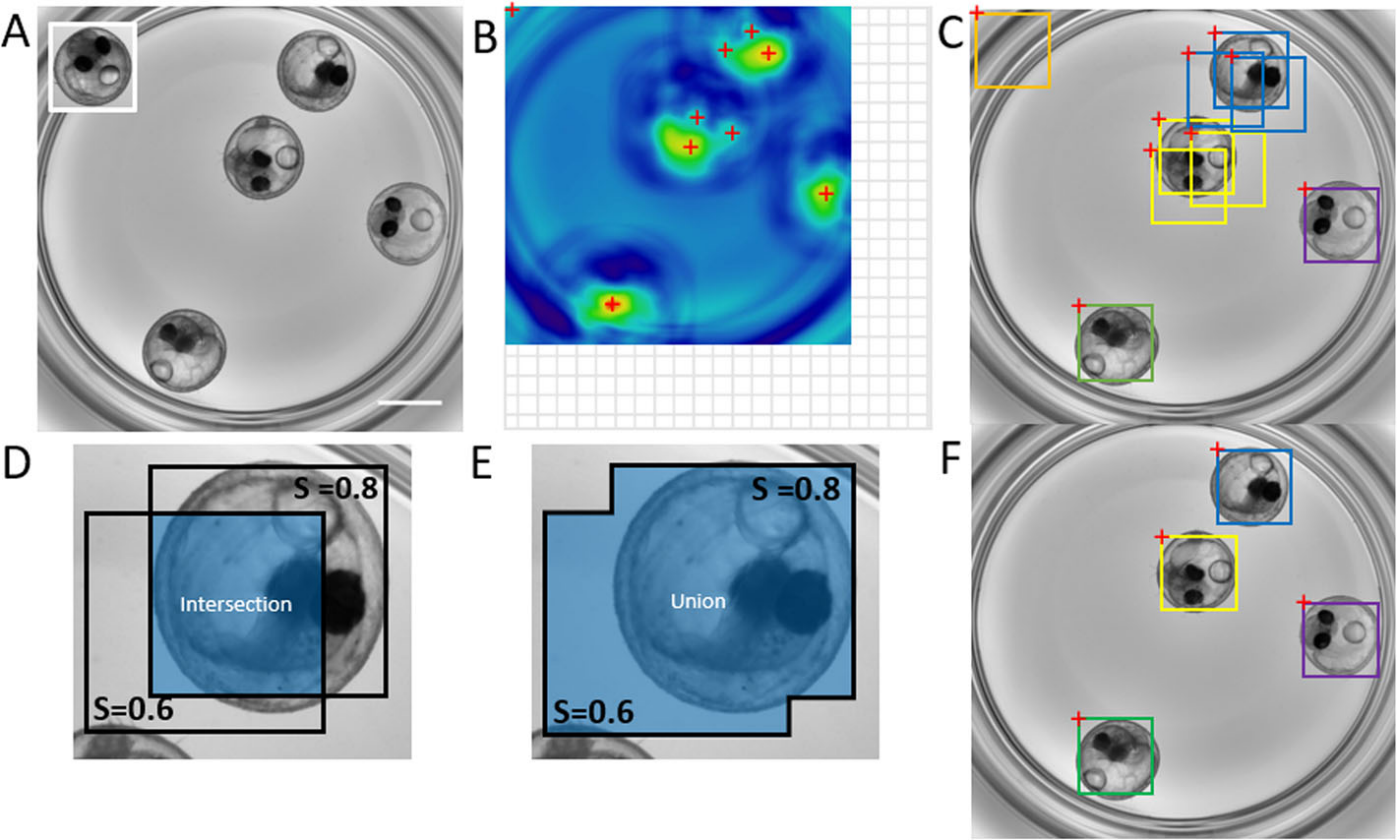
Multi-template matching and Non-Maxima Suppression for the detection of randomly oriented and positioned medaka embryos. **a** Image in which the search is performed (2048 × 2048 pixels - scale bar: 1 mm) and template as inset (400 × 414 pixels). The search was performed with a set of templates (original template, vertical and horizontal flip, each rotated by 90°, 180° and 270°). Parameters for the detection: score type: 0-mean normalized cross-correlation, *N* = 4 expected objects per image, score threshold: 0.35, maximal overlap between bounding boxes: 0.25. **b** One of the derived correlation maps from A: red crosses indicate possible local maxima before Non-Maxima Suppression (NMS). The grid area indicates the smaller size of the correlation map compared to the image in which the search is performed as explained in Additional file 13. **c** Bounding boxes associated to the maxima shown in **b** and overlaid on the searched image. Colours are highlighting overlapping bounding boxes. The bounding box dimensions are identical to the dimensions of the template used for the search. **d**, **e** Preventing overlapping detections by NMS. Shown are 2 overlapping bounding boxes predicting possible object locations. Each predicted location is associated to a probability score *S* to contain an object. The ratio between the intersection (**d**) and the union (**e**) area of the bounding boxes (Intersection over Union or IoU) is computed to decide whether the 2 overlapping bounding boxes are likely to predict the location of the same object (IoU close to 1) or the locations of distinct objects that are close to each other (IoU close to 0). For a detailed description of Non-Maxima Suppression see Additional file 13. **f** Yielded object detections after NMS with a maximal IoU of 0.25, to return the N_objects = 4 best detections


To predict the position of a template within a target image (Figs. 1a, 2a), the algorithm first computes a correlation map using the function *matchTemplate* from OpenCV (Figs. 1b, 2b). If information about the approximate position of the object is known a priori, the computation can be limited to a user-defined rectangular search region, which significantly speeds up the execution by reducing image size (Fig. 1a, b; Additional file 7: Figure S4, Additional file 9: Figure S6, Additional file 11: Figure S8 and Additional file 1: Movie S1). In this case, the image is first cropped to the search region before the computation of the correlation map, and the position of the predicted bounding boxes are recalculated for the initial image shape.

**Additional file 1: Movie S1.** Installation and single object detection. Available on YouTube at https://youtu.be/KlzIqSG5XBU.

Template matching performs the search by translation of the template over the image, i.e. it will find objects that show a similar orientation as in the template. To maximize the capacity of object-detection, our implementation allows to provide a set of templates to be searched (e.g. additional object perspectives, scales), or initial templates can be transformed by selecting additional flipping and rotation in the plugin interface. Using templates representing different objects, simultaneous object-detection for different categories can be performed, e.g. for classification of the detected regions (Additional file 11: Figure S8 and Additional file 12: Figure S9).

Detection of multiple objects from a single template search, but especially from multiple consecutive template searches can lead to overlapping detections when a simple ranking of corresponding correlation scores is performed (Fig. 2c). These redundant detections need to be recognized and eliminated in order to enable genuine and robust multi-template matching. To address this, we developed a custom strategy combining maxima finding in correlation maps, followed by NMS [15, 16], which excludes redundant detections based on a user-defined degree of overlap between predicted bounding-boxes (Fig. 2d, e, f). The detailed procedure for maxima detection and NMS can be found in Additional file 13.

The parameters for the detection (template rotation/flipping, expected number of objects, score type and if *N* > 1, threshold on the score and maximal overlap between predicted locations) are specified via a graphical user interface in both Fiji and KNIME (Additional file 5: Figure S2A and Additional file 6: Figure S3B). The detected regions are returned as rectangular regions of interest (ROI) in Fiji and as part of a mask image in KNIME, along with a result table listing the name of the template, the score and the coordinates for each detection (Additional file 5: Figure S2B and Additional file 6: Figure S3C). The tools are intuitively accessible and demand no programming experience. Importantly, the Fiji plugin is macro-recordable, and can thus be readily integrated in custom image processing workflows (see Additional file 3 for 2-step template matching).

## Results

We successfully tested our pipelines with whole organism screening datasets, e.g. for the detection of organs like head, trunk and eyes in oriented zebrafish larvae [17, 18] (Fig. 1, Additional file 7: Figure S4, Additional file 9: Figure S6 and Additional file 11: Figure S8, Additional file 1: Movie S1), or for the localization of multiple randomly oriented and positioned medaka embryos [19, 20] (Fig. 2, Additional file 10: Figure S7 and Additional file 2: Movie S2). The specificity of template matching for head detection in aligned zebrafish larvae was tested within full frame images and within a search region (Additional file 7: Figure S4). The implemented method performs robustly in the presence of experimental variability, such as slight changes of specimen morphology (Additional file 7: Figure S4C, e.g. well C7), altered orientation (Additional file 7: Figure S4C, e.g. well B8), partial occlusion (Additional file 7: Figure S4C, e.g. well E10) or noise (Additional file 8: Figure S5). In contrast, detection of comparably small structures by template matching within large heterogenous images can be challenging due to the increased likelihood of false detections. This can be largely improved using search regions (Additional file 9: Figure S6), or by successive template matching detections to first robustly detect an object and then the subregions within the object. For example, we illustrate the robust localization of zebrafish eye regions within previously detected head region using a custom 2-step template matching Fiji macro (Additional file 9: Figure S6B, C, Additional file 3).


**Additional file 2: Movie S2.** Multiple object detection. Available on YouTube at https://youtu.be/-PoZihjJIjQ.

Our implementation relies on the OpenCV library which provides a rapid computation of the correlation map (about 0.45 s/image in Fiji with a 188 × 194 template and 2048 × 2048 image) on a laptop with an intel i7-7500U CPU (2.7GHz) and 16 Gb of RAM. This computation can be even faster if a search region is provided (0.06 s/image with a 1820 × 452 pixels search area as in Fig. 1a, b, see also Additional file 7: Figure S4D and Additional file 9: Figure S6D).

In contrast to other template matching implementations, multi-template matching enables the robust detection of multiple objects displaying different intensity patterns by searching for several templates (e.g. additional geometrical transformations or different object categories) (Fig. 2, Additional file 10: Figure S7). The corresponding searches are consecutively executed for each template; thus, accuracy is achieved at the expense of computation time (Additional file 9: Figure S6B, S6D and Additional file 10: Figure S7B, S7C). Using templates representing different objects, simultaneous object-detection for different categories e.g. for classification of the detected regions can be performed. For instance, the multi-template approach could be used to score certain organ and tissue-specific phenotypes in whole-organism screening, as demonstrated by the categorization of zebrafish embryonic kidney phenotypes in a benchmark dataset [21, 22] (Additional file 12: Figure S9).

Previous implementations of template matching [14, 23, 24] do not allow to use different templates for object detections, neither do they provide a way to prevent multiple detections of the same object, which motivated our implementation of a NMS strategy. A comparison to previous template matching tools available in common end-user bioimage analysis software is given in Table 1. Importantly, we aimed at keeping our implementation simple and accessible to researchers of any background. Therefore, we limited the number of parameters to a minimum, and provide extensive documentation in the form of a wiki and online video tutorials (see links in Additional files 1 and 2). For the python implementation, we host several jupyter notebook tutorials on GitHub that can be executed directly in a web-browser without any installation using Binder [26].

**Table 1:** Comparison of end-user implementations of template matching, available in common bioimage analysis software

Name	Availability	Open source	Doc.	Multiple detections	Non-Maxima Suppression	Search region(s)	Transformations	Multiple templates	Reference
Template matching	Fiji	✓	✗	✗	✗	✗	✗	✗	[14]
MatchTemplate	CellProfiler	✓	✓	✗	✗	✗	✗	✗	[25]
Template Matching	ImageJ	✓	✓	✓	✗	✗	✗	✗	Link
cvMatch_Template	ImageJ	✓	✓	✓	✗	✗	✗	✗	[24]
Pattern Matching	NI Vision Development	✗	✓	✓	?	✓	✓	✗	Link
Multi-Template Matching	Fiji, Python, KNIME	✓	✓	✓	✓	✓	✓	✓	This paper

The table lists and compares available implementations and associated functionalities of template matching in common end-user software (Doc.: Documentation available,). The column *Transformations* corresponds to optional search with additional templates, generated by geometric transformation (e.g. flipping, rotations) of the initial templates. Link 1: https://imagej.nih.gov/ij/plugins/template-matching.html - Link 2: http://www.ni.com/example/30594/en/

## Discussion

Template matching for object-localization is easy to handle and understand by non-experts. It relies on normalized grey level comparisons between a template image and successive image patches from a sliding window. It considers the full intensity pattern of the template image that usually includes object and surrounding context information. This intensity signature has a powerful discriminative power, even upon occlusion of the object or noise in the image, and the normalized score renders the detection robust against shifted illumination conditions (Additional file 7: Figure S4 and Additional file 8: Figure S5). However, template matching is limited to searching for a single intensity pattern, rendering it sensitive to changes in orientation or perspective of objects. In our approach, we overcome this limitation of existing implementations by combining results from multiple template images to increase the range of detectable intensity patterns. We then used a custom NMS strategy based on the degree of overlap between predicted bounding boxes to prevent redundant detections of a given object. Using the overlap for NMS is the most generic way to filter detections by bounding boxes of possibly different sizes and aspect-ratios.

Template images are typically generated by cropping objects of interest from source images, but we also provide the automated generation of additional templates through geometrical transformations. To ensure user-friendliness, we restricted the optional template transformations to flipping and rotation, which represent the common object-transformations expected in microscopy images. However, the tool accepts an arbitrary number of template images representing other potential transformations such as scaling or distortion. While our implementation accepts discrete rotation angle values, rotational search should be balanced with required detection efficiency to keep the overall number of generated templates and thus computation time low. In this study, image examples originating from zebrafish screening studies [17] were obtained using sample mounting strategies to constrain the rotational orientation of specimen [18], thus preventing the need for rotational search and providing datasets that can be rapidly profiled with template matching approaches. A number of methods have been reported in the literature to prevent repeated searches with rotated templates [27–32]. However, the complexity of these approaches limits their usability by non-experts, or their implementation is not publicly available.

Template matching can also be used for supervised classification, e.g. for nearest-neighbour search based on a set of annotated templates. This method has the advantage that it does not require any pre-processing of the image, or computation and selection of features. However, the quality of the classification depends heavily on the choice of the templates, and the method is rotation and scale sensitive.

Besides the number of templates, the computation time is a function of image and template sizes. As illustrated in the result section (Additional file 7: Figure S4D, Additional file 9: Figure S6D, Additional file 11: Figure S8D and Additional file 1: Movie S1), the processing speed can be drastically increased by limiting the analysis to a search region in which the object of interest is expected. Alternatively, the search could be performed with downscaled versions of the image and templates followed by rescaling and placement of bounding boxes. Yet, because downscaling degrades the searched intensity pattern, this approach may lead to non-specific and potentially shifted detections; therefore, we do not provide this option in the Fiji and KNIME versions. However, advanced users can refer to the online tutorial of the python implementation for an example of how to use downscaling to accelerate the detection (see Additional file 13).

The current implementation is mainly targeted towards analysis of single channel grayscale images. RGB image data can be used as input but is automatically converted to grayscale average projections. Further major developments would be required to expand the tool to also consider colour information of objects of interest. The template matching originates from machine vision applications for the automated inspection of two-dimensional image data. Nevertheless, for certain applications it could also be used to search for the most probable XYZ positions of an object in volumetric data, provided that objects can be robustly discriminated between single z-slices.

## Conclusion

We demonstrate a novel implementation of template matching for object-localization in 2D images using multiple template images, thus drastically improving overall sensitivity and applicability of the method. Our implementation requires only few parameters and is easy to handle by non-expert users via an intuitive graphical interface in Fiji and KNIME. Advanced users are provided with a dedicated python implementation to create custom workflows. We demonstrate the utility of multi-template matching for the detection and possible classification of entire or partial biological specimen in microscopy images. Multi-template matching is highly flexible and can be used as a general image-analysis step for a multitude of applications and samples, as its detection potential mainly depends on the choice of appropriate template image. The usage of multiple template images for the search typically improves detection capacity but increases the computation time accordingly. To improve computing efficiency, the parallelization of template searches or GPU computing with OpenCV could be explored. Finally, the demonstrated template matching tools could also facilitate feedback microscopy applications by interfacing it with the control software of automated microscopes, thus enabling the automated acquisition of ROI for tracking or automated zooming-in on target structures without manual intervention [21, 23].

## Availability and requirements

**Project name:** Multi-Template Matching

**Project home page:** https://multi-template-matching.github.io/Multi-Template-Matching/

**Operating system(s):** Platform independent

**Programming languag**e: Python

**Other requirements:** Fiji with minimum Image 1.52o, IJ-OpenCV 1.2.1

**License:** GPL v3.0

**Any restrictions to use by non-academics:** Any derived work should be under GPL-compatible license

## Supplementary information

**Additional file 3: Macro.** 2step-TemplateMatching.ijm, available on the GitHub repository at https://github.com/multi-template-matching/MultiTemplateMatching-Fiji/blob/master/Fiji.app/scripts/Plugins/Multi-Template-Matching/2step-TemplateMatching.ijm. See *Supplementary Material.pdf* and *Supplementary Figures.pdf* for further information.

**Additional file 4: Figure S1.** Flowchart of the implemented multi-template matching. The chart illustrates the sequential execution of the tool, for correlation-based score. For difference-based score, the pipeline is identical except that a difference map is computed, minima are detected instead of maxima and the lowest minima are returned. (IoU: Intersection over Union).x

**Additional file 5: Figure S2.** Implementation in Fiji. (A) Graphical user interface for the plugin *“Template Matching Image”* with: (1) Dropdown menu to select the template image of the object of interest. The template must be smaller than the image specified in 2, (2) dropdown menu to select an image (or stack of images) in which to search for the template, (3) tick-boxes to optionally generate additional templates by horizontal/vertical flipping of the initial template, (4) input field for rotation angles to generate additional templates by rotations of the initial and, if selected, flipped templates. The angles are specified in degrees with clockwise orientation and must be separated by commas, (5) dropdown menu to choose the score used for the computation of the score map (normalised square-difference, normalised cross-correlation or 0-mean normalised cross-correlation), (6) input field to specify the number of objects expected in the image, (7) input field to enter a score-threshold in the range 0–1. If the normalised square-difference is selected, only local minima with values below the threshold are returned. While for cross-correlation scores, maxima above this value are returned, (8) input field to specify the maximum value in range 0–1 for the intersection over union (IoU) between a pair of overlapping bounding boxes (Non-Maxima Suppression), (9) tick-box to select if the detected Regions Of Interest (ROI) should be added to Fiji ROI Manager, (10) tick-box to specify if the result table should be displayed at the end of the execution. Parameters 7 and 8 are only required if several objects are expected in each image. (B) Outputs of the plugin with (1) result table with each row containing the names of the image and template, the prediction score and coordinates of the top left corner and centre of the predicted bounding box, and (2) the detected ROI appended to the ROI Manager and highlighted on the image (yellow).

**Additional file 6: Figure S3.** Implementation in KNIME. (A) Screenshot of the KNIME workflow. The template and images are provided in the *Image Reader* nodes on the left side, the processing happens in the central metanode called ‘*Multi-Template Matching*’ containing a python node calling the python implementation. The parameters for multi-template matching can be configured via a graphical user interface (see B) by right clicking on the node. The predicted locations can be visualised in the *Interactive Segmentation View* node on the right side (as shown in C). A result table containing the bounding box position, dimension and correlation score is also returned (Table view node, output not shown). (B) Graphical user interface of the central ‘*Multi-Template Matching*’ metanode for the configuration of the detection parameters, similarly to the Fiji implementation (see Additional file 5: Figure S2A). (C) Predicted locations as viewed in the *Interactive Segmentation View* node. The predicted locations are composed into a mask and overlaid on the image.

**Additional file 7: Figure S4.** Template matching for head region detection in oriented zebrafish larvae. (A) Single template (188 × 194 pixels, no additional transformation) and image (2048 × 2048 pixels, scale bar: 1 mm) in which the search is performed. The orange rectangle shows the optionally used restricted search region (1820 × 452 pixels). Parameters for the detection: score type: 0-mean normalised cross-correlation – *N* = 1 expected object per image. (B) Result of the detection for *N* = 96 images, with and without search region (both 100% detection rate). (C) Montage of detected zebrafish larval head regions within a 96 well plate (as in Fig. 1c). (D) Mean computation time per image (error bars show standard deviation) for the different conditions as in B using the same computing hardware as in the main text. Prior information about the position of the sample within the field of view (e.g. due to standardized sample mounting) can be used to specify a search region, drastically accelerating the computation and reducing the chance of incorrect predictions.

**Additional file 8: Figure S5.** Multi-template matching is robust to noise. (A) Original image (2048 × 2048 pixels). (B) Image as in A corrupted with artificial noise (normally distributed random noise – mean:0, standard deviation:50). (C, D) Result of multi-template matching for respectively A and B. The template used is a crop of the specimen in the middle of image A (hence a correlation score of 1 for the first row of Table C). Parameters for the detection: rotation of the template: 90,180° - score type: 0-mean normalised cross-correlation - *N* = 4 expected objects per image – score threshold: 0.3 – maximal overlap between bounding boxes: 0.3.

**Additional file 9: Figure S6.** Multi-template matching for eye-region detection in oriented zebrafish larvae. (A) Templates (108 × 76 pixels) and image in which the search is performed (2048 × 2048 pixels, scale bar: 1 mm). Orange rectangle indicates optional search region (1820 × 452 pixels), and blue dotted rectangle the head-region template used for 2-step template matching (see B and D). Parameters for the detection: Vertical flipping of the templates (only if *FlipV* indicated), score type: 0-mean normalized cross-correlation, *N* = 2 expected objects per image, score threshold: 0.5, maximal overlap between bounding boxes: 0.25. For 2-step template matching the search region in orange is used for the 1st step (head-detection with a single head template, N = 1 expected object), then a single eye template is used with flipping for the detection of the eyes within the previously detected head region. (B) Result of the detections for *N* = 94 images. *2 eyes/1 eye/No eyes* in figure legend refer to the outcome of eye-region detection in each larva. Vertical flipping of the templates readily increases the number of genuine matches. The 2-step template matching approach (search of template within a previously identified ROI) offers the best results and is recommended for more challenging template images (see Additional file 3). (C) Montage of the eye regions detections (yellow) for the 2-step matching approach as in B and D. Specimen in well B8 and F7 are excluded from the count in B as they are not dorsally oriented. (D) Mean computation time per image (error bars show standard deviation) for the different conditions (as in B) using the same hardware as in the main text.

**Additional file 10: Figure S7.** Multi-template matching for the localization of randomly oriented and positioned medaka embryos. (A) Initial template (410 × 420 pixels) and one of the images in which the search is performed (2048 × 2048 pixels, scale bar of 1 mm). The yellow bounding boxes indicate predicted locations when only the original template in A is used for the search, the green boxes indicate predicted locations when using a set of templates (original, horizontal and vertical flipping, rotation of the original and flipped templates by 90°,180° and 270°). Parameters for the detection: score type: 0-mean normalized cross-correlation, N = 4 expected objects per image, score threshold:0.35, maximal overlap between bounding boxes:0.25. (B) Result of the detections for 10 images each containing 4 embryos (i.e. 40 embryos in total) See detected region in D. (C) Mean computation time per image (error bars show standard deviation) for the different conditions using the same hardware as in the main text. The computation time for each image scales with the number of templates. (D) Montage of the detected regions for 10 images similar to A, each containing 4 embryos (1 column/image). The montage corresponds to the benchmark *“1 Template + transformations”* as in B and C. Yellow bounding boxes indicate the 2 detections classified as *Partial* in B.

**Additional file 11: Figure S8.** Multi-template matching for simultaneous head and trunk region detection in oriented zebrafish larvae. (A) Head (188 × 194 pixels) and trunk region (264 × 192 pixels) templates. Image (2048 × 2048 pixels, scale bar: 1 mm) in which the search is performed. The orange rectangle shows the optionally used restricted search region (1820 × 452 pixels). Parameters for the detection: Vertical flipping of the templates - score type: 0-mean normalised cross-correlation – N = 2 expected objects per image – score threshold: 0.6 – maximal overlap between bounding boxes: 0.35. (B) Result of the detection for *N* = 96 images, with and without search region. (C) Montage of the detected head regions in 96 zebrafish larvae when the search region is used. The head region was not detected in 3 specimens, 2 of them were not properly dorsally aligned. (see. Additional file 7: Figure S4C). (D) Montage of the detected trunk regions in 96 zebrafish larvae when the search region is used. When 2 trunks were detected in one image (instead of one trunk and one head), the trunk with the best score was used for the montage. Prior information about the position of the sample within the field of view (e.g. due to standardized sample mounting) can be used to specify a search region, drastically accelerating the computation and reducing the chance of incorrect predictions. (E) Mean computation time per image (N = 96 - error bars show standard deviation) for the different conditions as in B using the same computing hardware as in the main text.

**Additional file 12: Figure S9.** Using multi-template matching for phenotypes classification. (A) Manually annotated templates used for the classification of phenotypes of embryonic zebrafish kidneys. (B) Example of image to classify and (C) resulting correlation-scores for the 3 classes. In this case, the image is correctly classified as cystic. (D) Confusion matrix depicting the results for the classification of 167 annotated images (50 normal, 52 cystic, 65 long). The class *Cystic* and *Normal* are particularly well predicted. A number of *Long* were classified as *Cystic*, this can be expected as those 2 morphologies show similar elongated regions.

**Additional file 13. **Supplementary information. Supplementary Material for *Multi-Template Matching: a versatile tool for object-localization in microscopy images.*

## Data Availability

The image datasets used for this study are available on Zenodo (links below). - Dataset used for Fig. 1, Additional file 7: Figure S4, Additional file 9: Figure S6 and Additional file 11: Figure S8. Gehrig, Jochen. (2019). 3dpf zebrafish larvae, 96 well plate, Tg (wt1b:EGFP), dorsal view, ACQUIFER Imaging Machine [Data set]. Zenodo. 10.5281/zenodo.2650162 -. - Dataset used for Fig. 2, Additional file 8: Figure S5 and Additional file 10: Figure S7. Gierten Jakob & Gehrig Jochen. (2019). 102 hpf medaka embryos in 96 well plate (4 embryo/well) - brightfield - 2X magnification - ACQUIFER Imaging Machine (Version 1) [Data set]. Zenodo. 10.5281/zenodo.2650147

## Abbreviations

Doc: Documentation
IoU: Intersection over Union
NMS: Non-Maxima Suppression
ROI: Region of Interest
