# Correction to: Multi-template matching: a versatile tool for object-localization in microscopy images

**DOI:** 10.1186/s12859-021-04524-7

**Published:** 2022-01-04

**Authors:** Laurent S. V. Thomas, Jochen Gehrig

**Affiliations:** 1 Acquifer Is a Division of Ditabis, Digital Biomedical Imaging Systems AG, Pforzheim, Germany; 2 Centre of Paediatrics and Adolescent Medicine, University Hospital Heidelberg, Heidelberg, Germany

## Correction to: BMC Bioinformatics (2020) 21:44 10.1186/s12859-020-3363-7

Following the publication of the original article [1], the authors would like to update Additional file 3 in the supplementary information. The broken link and file have been corrected and updated.

The original article [1] has been corrected.

## Supplementary Information

**Additional file 3: Macro**. 2step-TemplateMatching.ijm, available on the GitHub repository at https://github.com/multi-template-matching/MultiTemplateMatching-Fiji/blob/master/Fiji/scripts/Plugins/Template_Matching/2step-TemplateMatching.ijm. See Supplementary Material.pdf and Supplementary Figures.pdf for further information.
