# Supplementary material for: Multi-template matching: a versatile tool for object-localization in microscopy images
Source: BMC Bioinformatics. 2020 Feb 5;21:44. doi: 10.1186/s12859-020-3363-7 (PMC7003318; doi:10.1186/s12859-020-3363-7)
Supplement: Supplementary file 4 — Additional file 4: Figure S1. Flowchart of the implemented multi-template matching. The chart illustrates the sequential execution of the tool, for correlation-based score. For difference-based score, the pipeline is identical except that a difference map is computed, minima are detected instead of maxima and the lowest minima are returned. (IoU: Intersection over Union).x [file 12859_2020_3363_MOESM4_ESM.pptx]

## Slide 1
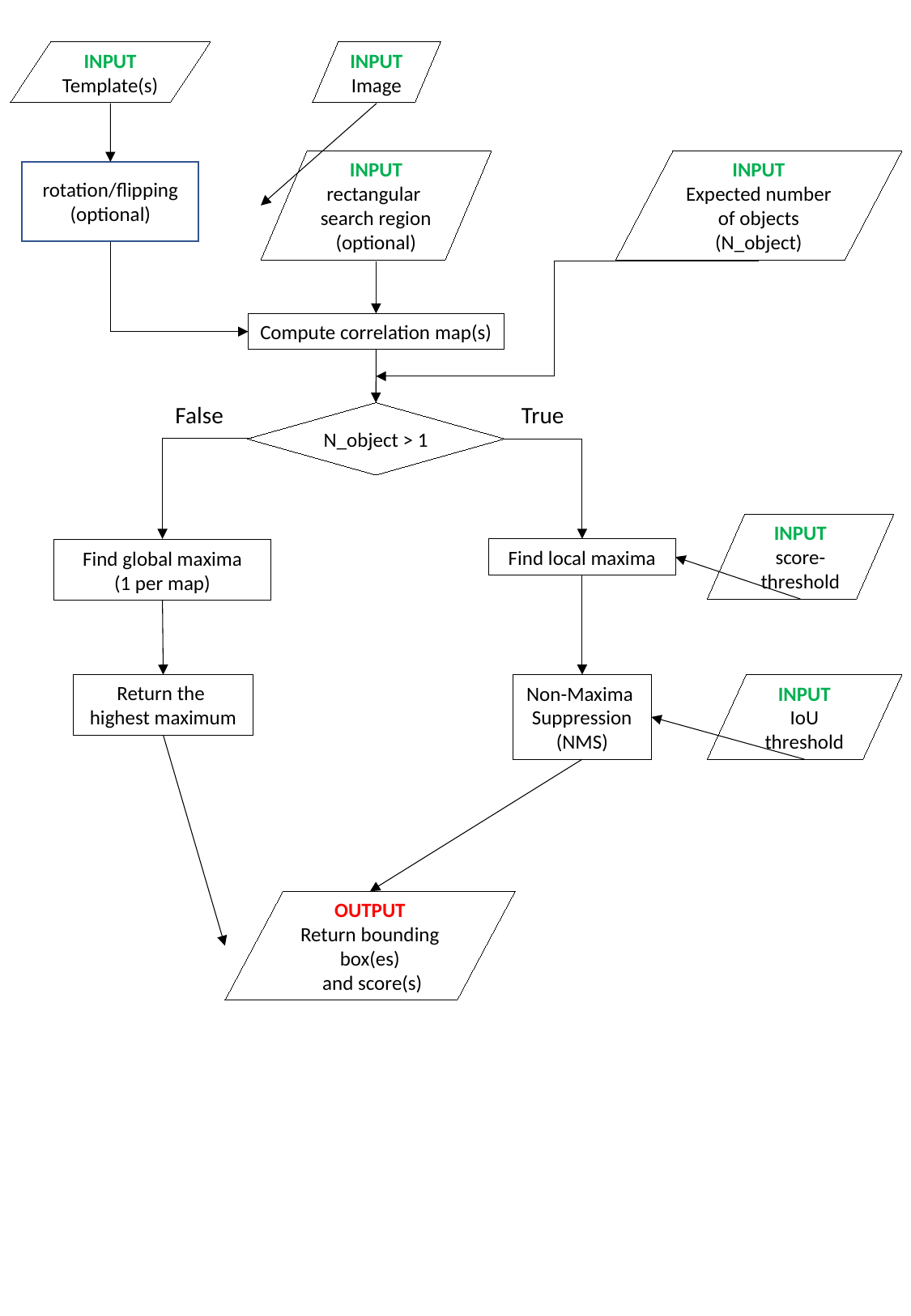

INPUT
Template(s)
Compute correlation map(s)
INPUT
Image
INPUT
rectangular
search region
(optional)
INPUT
Expected number
of objects (N_object)
rotation/flipping
(optional)
True
False
N_object > 1
INPUT
score-threshold
Find local maxima
Non-Maxima
Suppression (NMS)
Find global maxima
(1 per map)
Return the
highest maximum
INPUT
IoU threshold
OUTPUT
Return bounding box(es)
 and score(s)
