# Supplementary figures and images for: Multi-template matching: a versatile tool for object-localization in microscopy images
Source: BMC Bioinformatics. 2020 Feb 5;21:44. doi: 10.1186/s12859-020-3363-7 (PMC7003318; doi:10.1186/s12859-020-3363-7)

## Slide 1
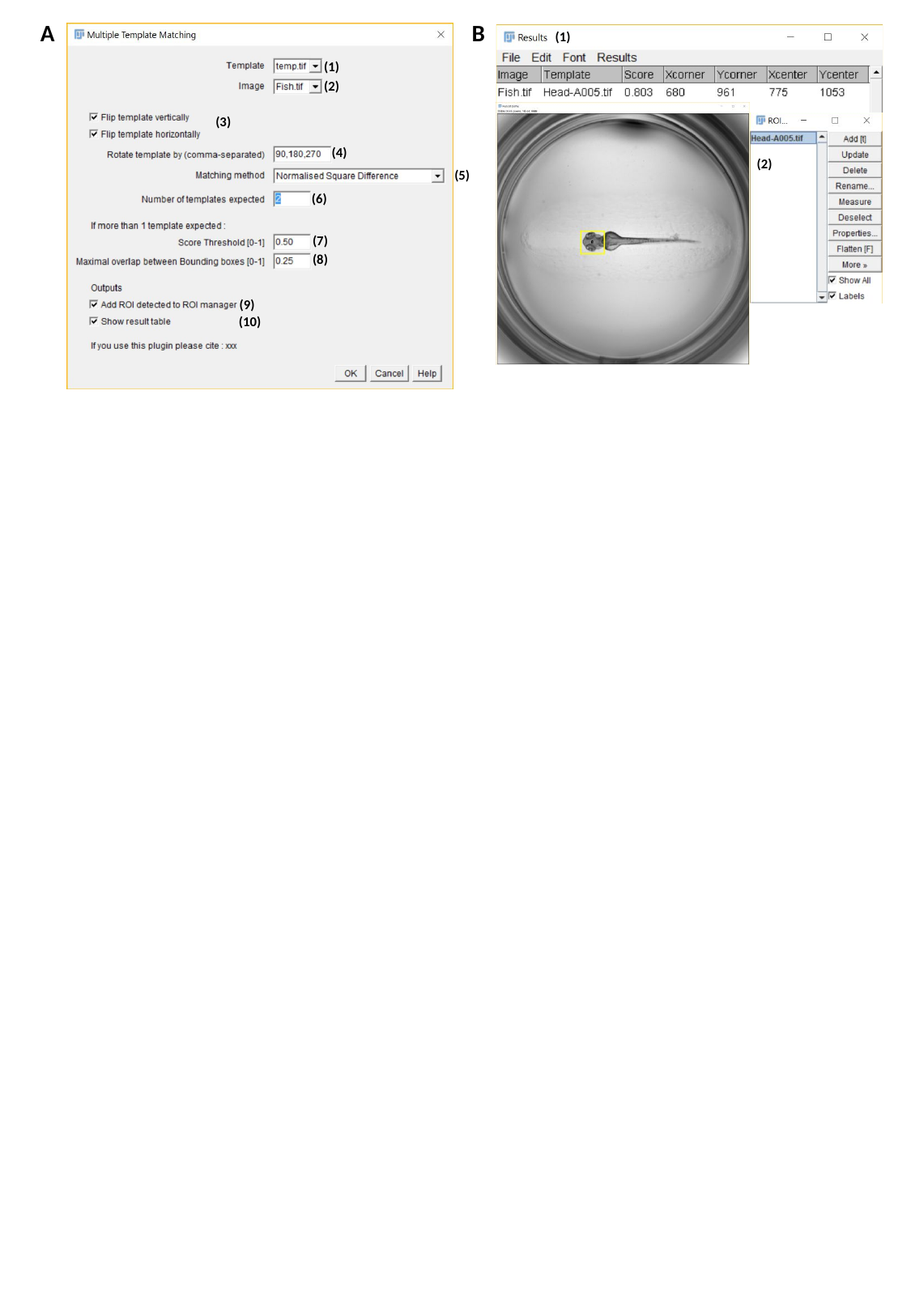

B
A
(1)
(2)
(3)
(4)
(5)
(6)
(7)
(8)
(9)
(10)
(1)
(2)

Supplement: Supplementary file 5 — Additional file 5: Figure S2. Implementation in Fiji. (A) Graphical user interface for the plugin “Template Matching Image” with: (1) Dropdown menu to select the template image of the object of interest. The template must be smaller than the image specified in 2, (2) dropdown menu to select an image (or stack of images) in which to search for the template, (3) tick-boxes to optionally generate additional templates by horizontal/vertical flipping of the initial template, (4) input field for rotation angles to generate additional templates by rotations of the initial and, if selected, flipped templates. The angles are specified in degrees with clockwise orientation and must be separated by commas, (5) dropdown menu to choose the score used for the computation of the score map (normalised square-difference, normalised cross-correlation or 0-mean normalised cross-correlation), (6) input field to specify the number of objects expected in the image, (7) input field to enter a score-threshold in the range 0–1. If the normalised square-difference is selected, only local minima with values below the threshold are returned. While for cross-correlation scores, maxima above this value are returned, (8) input field to specify the maximum value in range 0–1 for the intersection over union (IoU) between a pair of overlapping bounding boxes (Non-Maxima Suppression), (9) tick-box to select if the detected Regions Of Interest (ROI) should be added to Fiji ROI Manager, (10) tick-box to specify if the result table should be displayed at the end of the execution. Parameters 7 and 8 are only required if several objects are expected in each image. (B) Outputs of the plugin with (1) result table with each row containing the names of the image and template, the prediction score and coordinates of the top left corner and centre of the predicted bounding box, and (2) the detected ROI appended to the ROI Manager and highlighted on the image (yellow). [file 12859_2020_3363_MOESM5_ESM.pptx]

## Slide 1
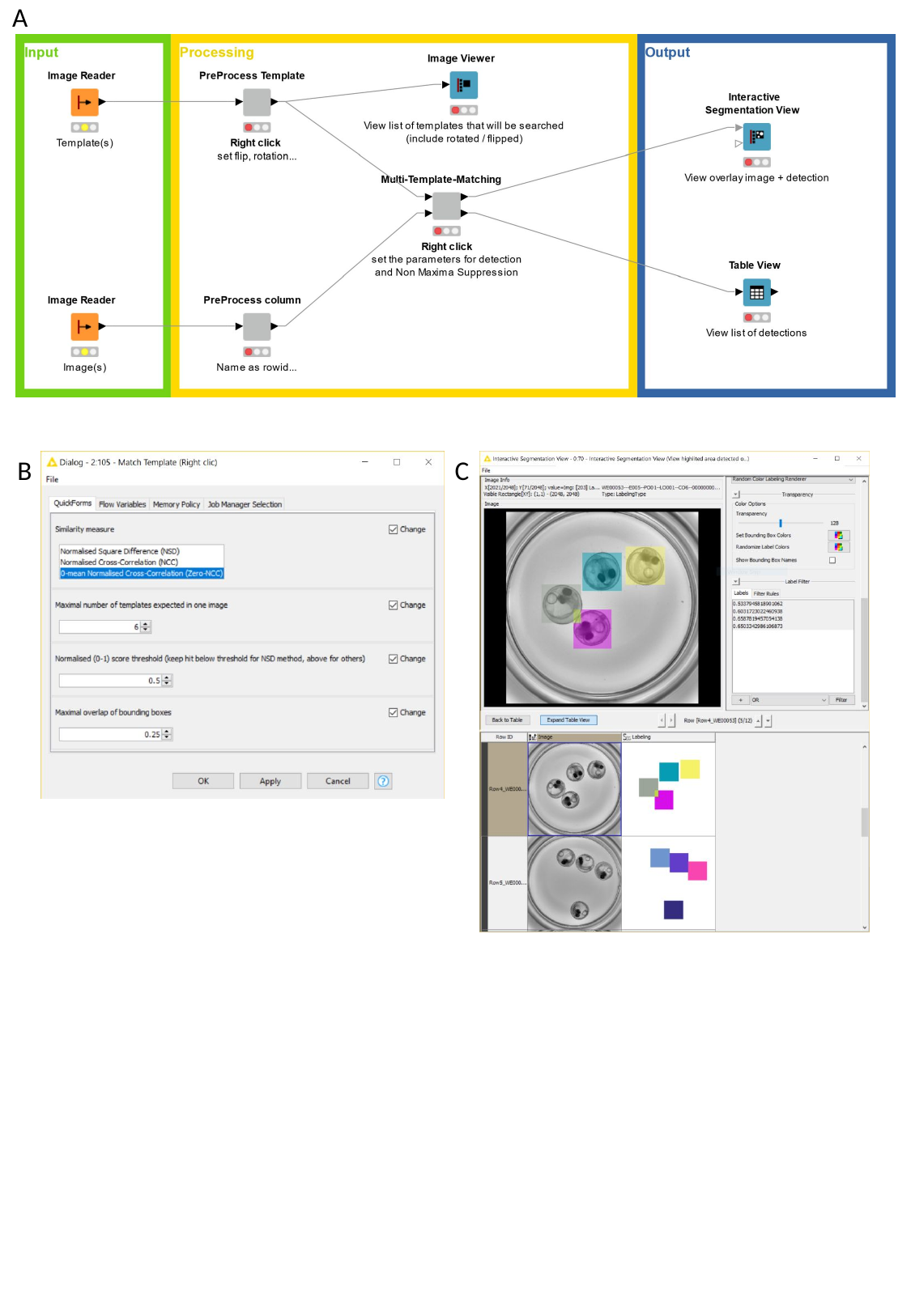

A
B
C

Supplement: Supplementary file 6 — Additional file 6: Figure S3. Implementation in KNIME. (A) Screenshot of the KNIME workflow. The template and images are provided in the Image Reader nodes on the left side, the processing happens in the central metanode called ‘Multi-Template Matching’ containing a python node calling the python implementation. The parameters for multi-template matching can be configured via a graphical user interface (see B) by right clicking on the node. The predicted locations can be visualised in the Interactive Segmentation View node on the right side (as shown in C). A result table containing the bounding box position, dimension and correlation score is also returned (Table view node, output not shown). (B) Graphical user interface of the central ‘Multi-Template Matching’ metanode for the configuration of the detection parameters, similarly to the Fiji implementation (see Additional file 5: Figure S2A). (C) Predicted locations as viewed in the Interactive Segmentation View node. The predicted locations are composed into a mask and overlaid on the image. [file 12859_2020_3363_MOESM6_ESM.pptx]

## Slide 1
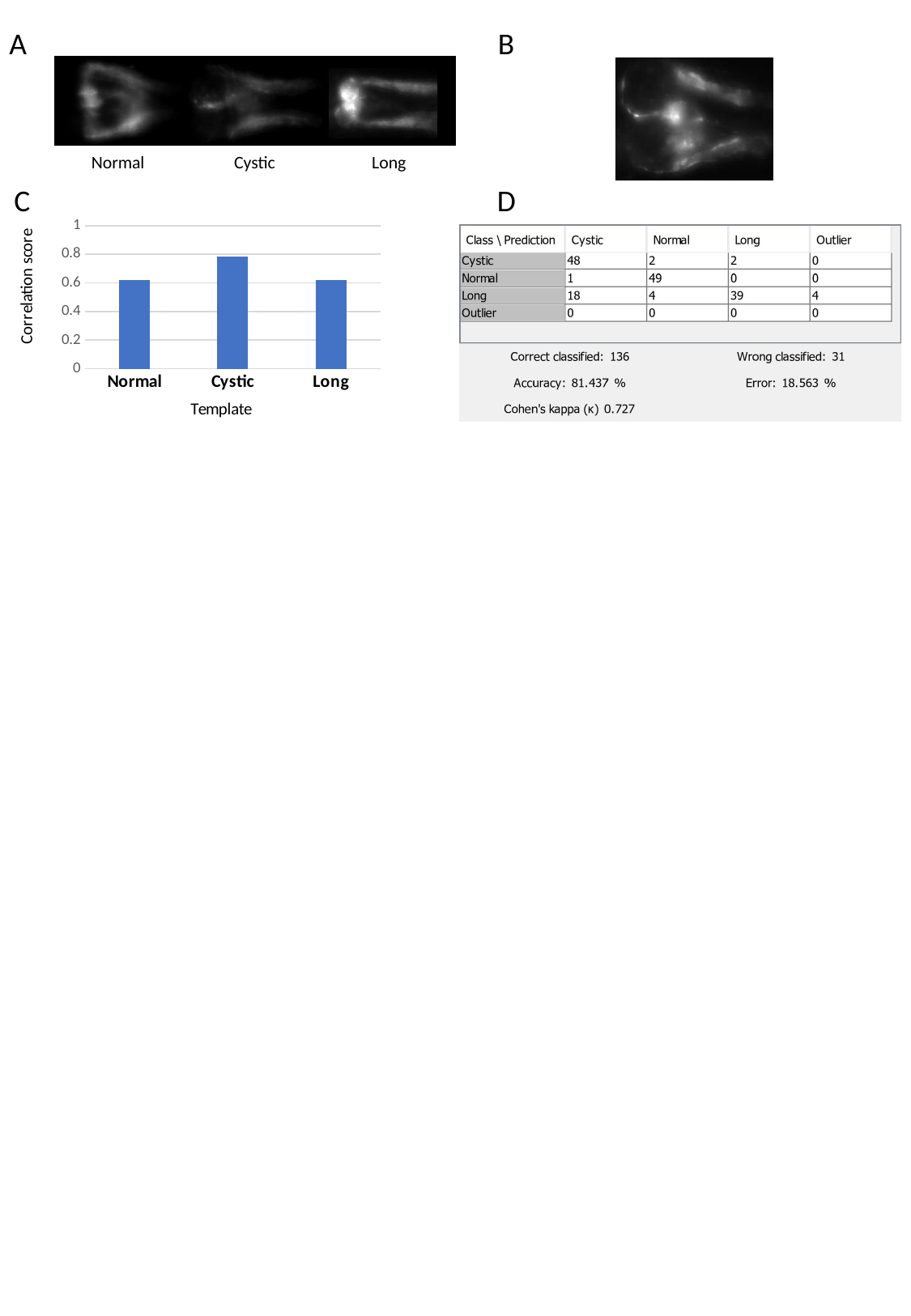

A
B
Normal
Cystic
Long
C
D
### Chart
| Category | |
|---|---|
| Normal | 0.618562340736389 |
| Cystic | 0.784682750701904 |
| Long | 0.622472941875457 |

Supplement: Supplementary file 12 — Additional file 12: Figure S9. Using multi-template matching for phenotypes classification. (A) Manually annotated templates used for the classification of phenotypes of embryonic zebrafish kidneys. (B) Example of image to classify and (C) resulting correlation-scores for the 3 classes. In this case, the image is correctly classified as cystic. (D) Confusion matrix depicting the results for the classification of 167 annotated images (50 normal, 52 cystic, 65 long). The class Cystic and Normal are particularly well predicted. A number of Long were classified as Cystic, this can be expected as those 2 morphologies show similar elongated regions. [file 12859_2020_3363_MOESM12_ESM.pptx]
