# Supplementary material for: Multi-template matching: a versatile tool for object-localization in microscopy images
Source: BMC Bioinformatics. 2020 Feb 5;21:44. doi: 10.1186/s12859-020-3363-7 (PMC7003318; doi:10.1186/s12859-020-3363-7)
Supplement: Supplementary file 7 — Additional file 7: Figure S4. Template matching for head region detection in oriented zebrafish larvae. (A) Single template (188 × 194 pixels, no additional transformation) and image (2048 × 2048 pixels, scale bar: 1 mm) in which the search is performed. The orange rectangle shows the optionally used restricted search region (1820 × 452 pixels). Parameters for the detection: score type: 0-mean normalised cross-correlation – N = 1 expected object per image. (B) Result of the detection for N = 96 images, with and without search region (both 100% detection rate). (C) Montage of detected zebrafish larval head regions within a 96 well plate (as in Fig. 1c). (D) Mean computation time per image (error bars show standard deviation) for the different conditions as in B using the same computing hardware as in the main text. Prior information about the position of the sample within the field of view (e.g. due to standardized sample mounting) can be used to specify a search region, drastically accelerating the computation and reducing the chance of incorrect predictions. [file 12859_2020_3363_MOESM7_ESM.pptx]

## Slide 1
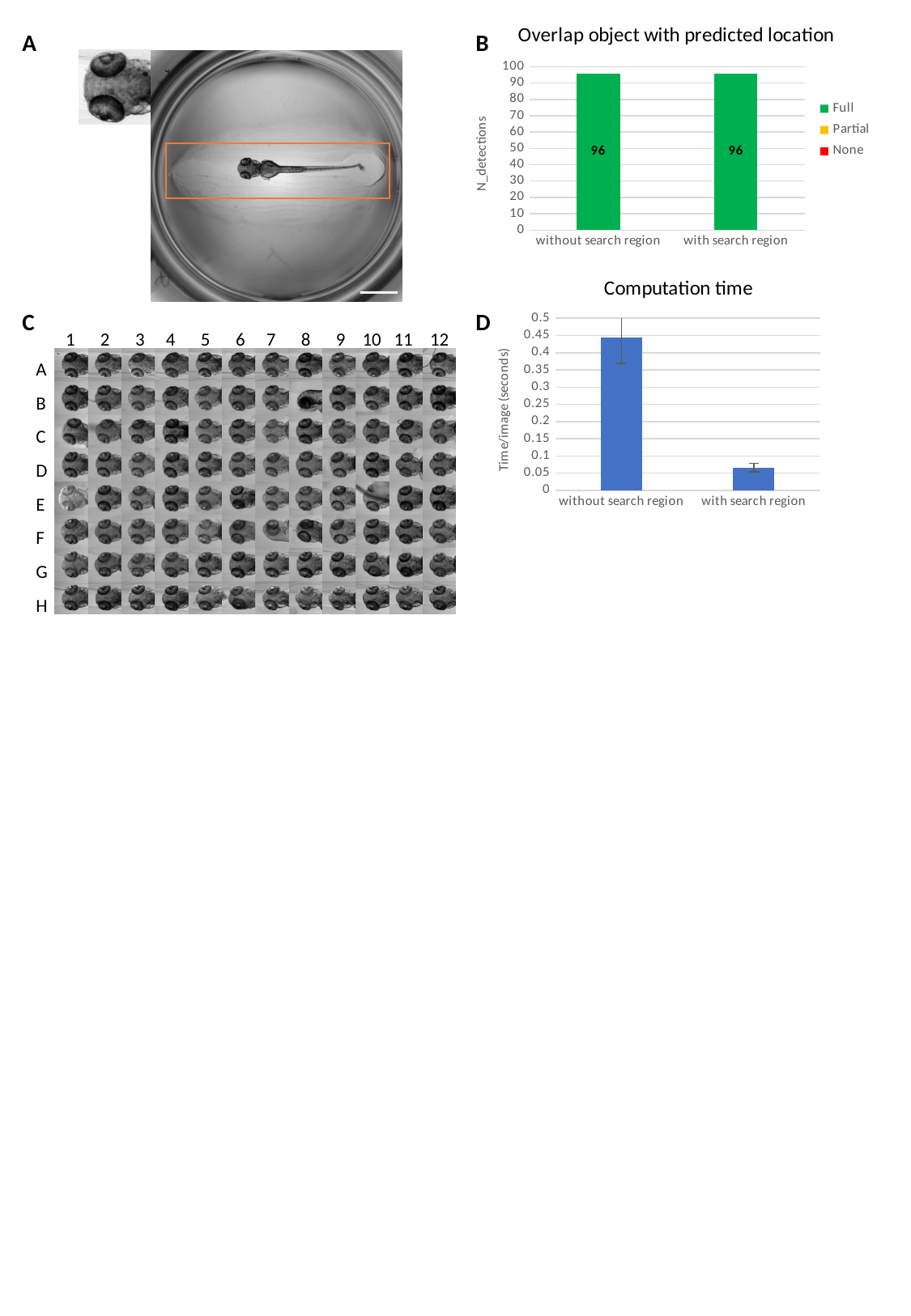

### Chart: Overlap object with predicted location
| Category | Full | Partial | None |
|---|---|---|---|
| without search region | 96.0 | 0.0 | 0.0 |
| with search region | 96.0 | 0.0 | 0.0 |A
### Chart: Computation time
| Category | time/image(s) |
|---|---|
| without search region | 0.444 |
| with search region | 0.066 |C
D
B
1 2 3 4 5 6 7 8 9 10 11 12
A
B
C
D
E
F
G
H
