# Supplementary material for: Multi-template matching: a versatile tool for object-localization in microscopy images
Source: BMC Bioinformatics. 2020 Feb 5;21:44. doi: 10.1186/s12859-020-3363-7 (PMC7003318; doi:10.1186/s12859-020-3363-7)
Supplement: Supplementary file 8 — Additional file 8: Figure S5. Multi-template matching is robust to noise. (A) Original image (2048 × 2048 pixels). (B) Image as in A corrupted with artificial noise (normally distributed random noise – mean:0, standard deviation:50). (C, D) Result of multi-template matching for respectively A and B. The template used is a crop of the specimen in the middle of image A (hence a correlation score of 1 for the first row of Table C). Parameters for the detection: rotation of the template: 90,180° - score type: 0-mean normalised cross-correlation - N = 4 expected objects per image – score threshold: 0.3 – maximal overlap between bounding boxes: 0.3. [file 12859_2020_3363_MOESM8_ESM.pptx]

## Slide 1
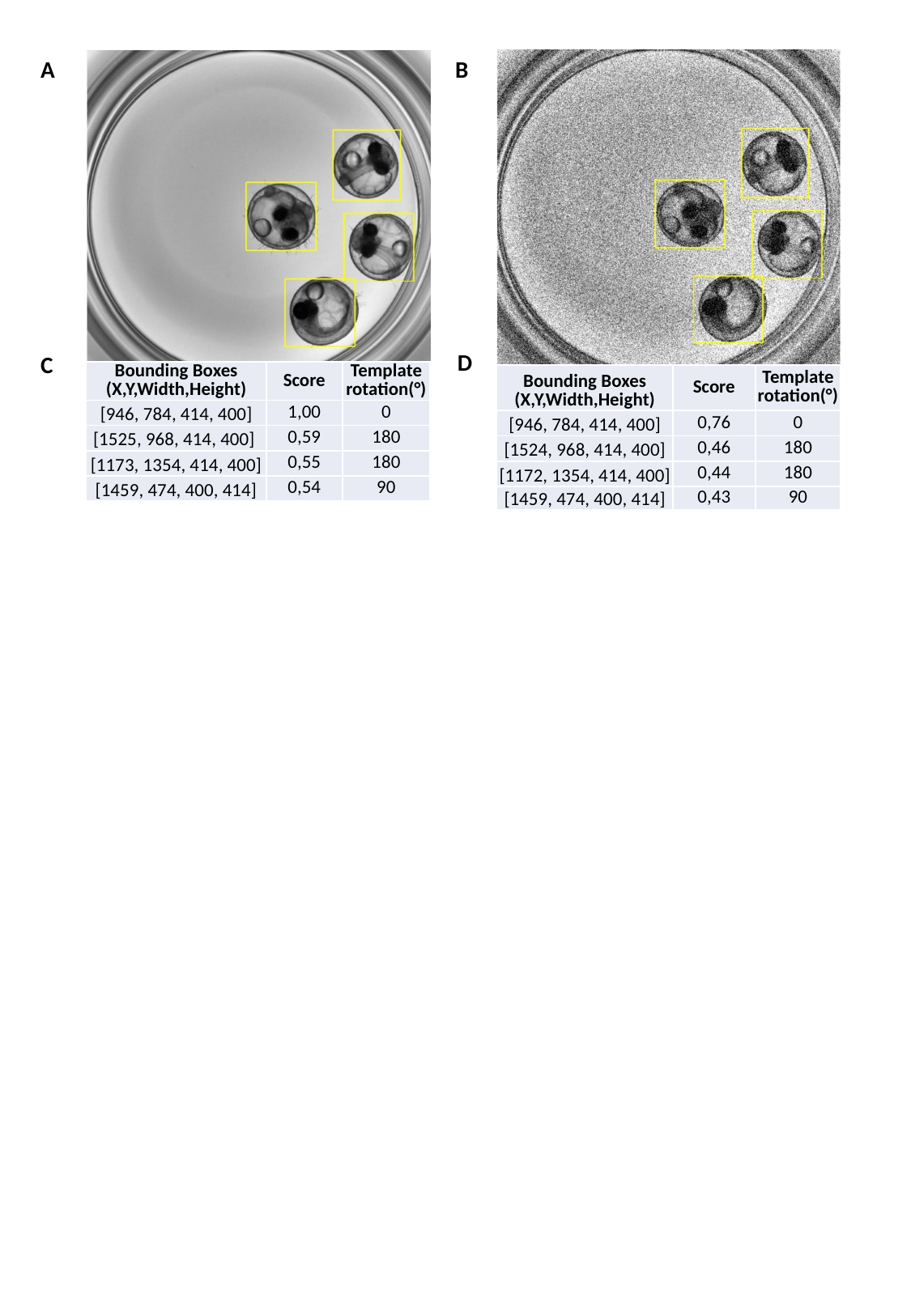

B
A
D
C
| Bounding Boxes (X,Y,Width,Height) | Score | Template rotation(°) |
| --- | --- | --- |
| [946, 784, 414, 400] | 1,00 | 0 |
| [1525, 968, 414, 400] | 0,59 | 180 |
| [1173, 1354, 414, 400] | 0,55 | 180 |
| [1459, 474, 400, 414] | 0,54 | 90 |
| Bounding Boxes (X,Y,Width,Height) | Score | Template rotation(°) |
| --- | --- | --- |
| [946, 784, 414, 400] | 0,76 | 0 |
| [1524, 968, 414, 400] | 0,46 | 180 |
| [1172, 1354, 414, 400] | 0,44 | 180 |
| [1459, 474, 400, 414] | 0,43 | 90 |
