# Supplementary material for: Multi-template matching: a versatile tool for object-localization in microscopy images
Source: BMC Bioinformatics. 2020 Feb 5;21:44. doi: 10.1186/s12859-020-3363-7 (PMC7003318; doi:10.1186/s12859-020-3363-7)
Supplement: Supplementary file 9 — Additional file 9: Figure S6. Multi-template matching for eye-region detection in oriented zebrafish larvae. (A) Templates (108 × 76 pixels) and image in which the search is performed (2048 × 2048 pixels, scale bar: 1 mm). Orange rectangle indicates optional search region (1820 × 452 pixels), and blue dotted rectangle the head-region template used for 2-step template matching (see B and D). Parameters for the detection: Vertical flipping of the templates (only if FlipV indicated), score type: 0-mean normalized cross-correlation, N = 2 expected objects per image, score threshold: 0.5, maximal overlap between bounding boxes: 0.25. For 2-step template matching the search region in orange is used for the 1st step (head-detection with a single head template, N = 1 expected object), then a single eye template is used with flipping for the detection of the eyes within the previously detected head region. (B) Result of the detections for N = 94 images. 2 eyes/1 eye/No eyes in figure legend refer to the outcome of eye-region detection in each larva. Vertical flipping of the templates readily increases the number of genuine matches. The 2-step template matching approach (search of template within a previously identified ROI) offers the best results and is recommended for more challenging template images (see Additional file 3). (C) Montage of the eye regions detections (yellow) for the 2-step matching approach as in B and D. Specimen in well B8 and F7 are excluded from the count in B as they are not dorsally oriented. (D) Mean computation time per image (error bars show standard deviation) for the different conditions (as in B) using the same hardware as in the main text. [file 12859_2020_3363_MOESM9_ESM.pptx]

## Slide 1
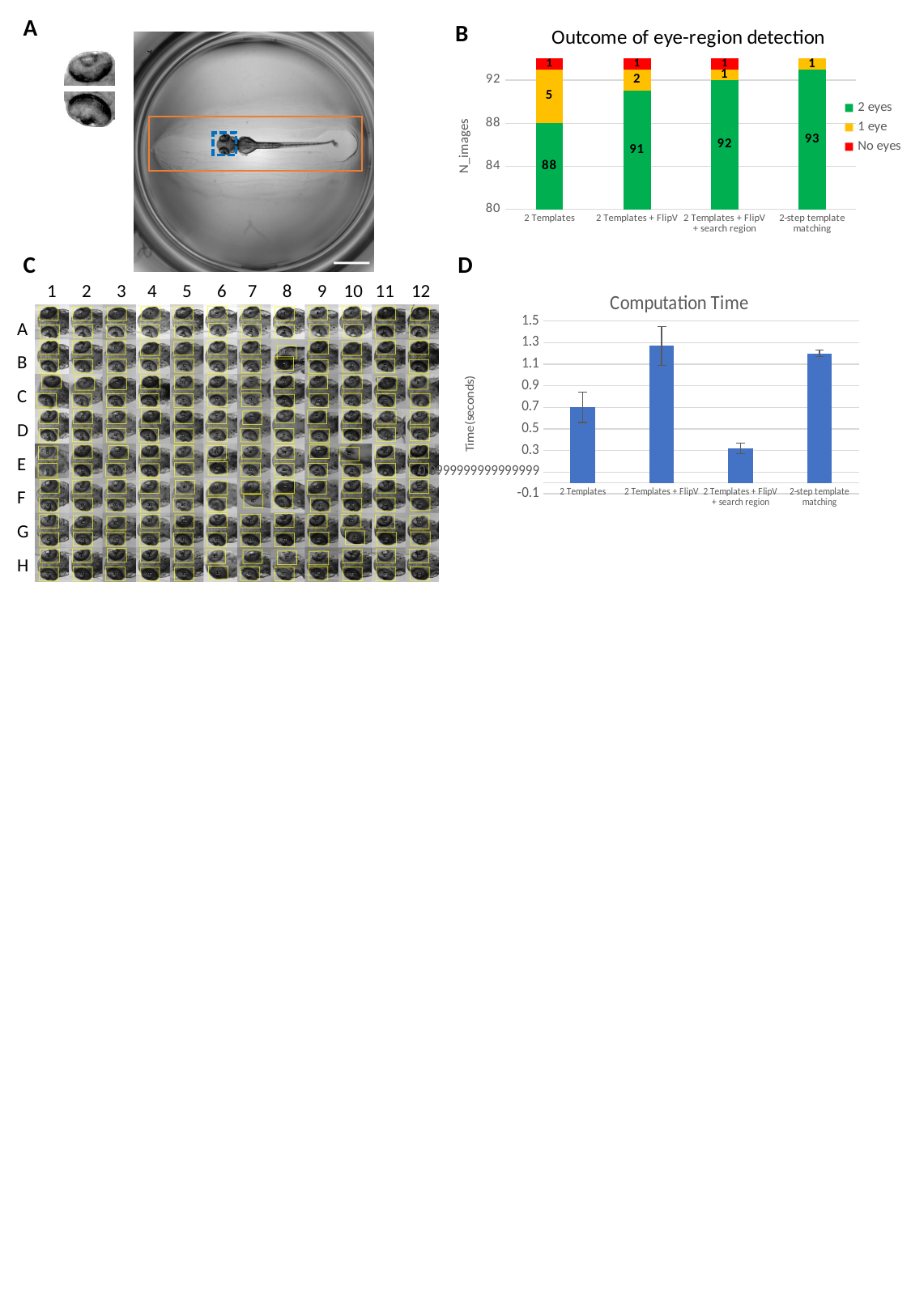

A
B
### Chart: Outcome of eye-region detection
| Category | 2 eyes | 1 eye | No eyes |
|---|---|---|---|
| 2 Templates | 88.0 | 5.0 | 1.0 |
| 2 Templates + FlipV | 91.0 | 2.0 | 1.0 |
| 2 Templates + FlipV + search region | 92.0 | 1.0 | 1.0 |
| 2-step template matching | 93.0 | 1.0 | 0.0 |
C
D
1 2 3 4 5 6 7 8 9 10 11 12
### Chart: Computation Time
| Category | |
|---|---|
| 2 Templates | 0.7 |
| 2 Templates + FlipV | 1.27 |
| 2 Templates + FlipV + search region | 0.32 |
| 2-step template matching | 1.2 |A
B
C
D
E
F
G
H
