# Supplementary material for: Multi-template matching: a versatile tool for object-localization in microscopy images
Source: BMC Bioinformatics. 2020 Feb 5;21:44. doi: 10.1186/s12859-020-3363-7 (PMC7003318; doi:10.1186/s12859-020-3363-7)
Supplement: Supplementary file 10 — Additional file 10: Figure S7. Multi-template matching for the localization of randomly oriented and positioned medaka embryos. (A) Initial template (410 × 420 pixels) and one of the images in which the search is performed (2048 × 2048 pixels, scale bar of 1 mm). The yellow bounding boxes indicate predicted locations when only the original template in A is used for the search, the green boxes indicate predicted locations when using a set of templates (original, horizontal and vertical flipping, rotation of the original and flipped templates by 90°,180° and 270°). Parameters for the detection: score type: 0-mean normalized cross-correlation, N = 4 expected objects per image, score threshold:0.35, maximal overlap between bounding boxes:0.25. (B) Result of the detections for 10 images each containing 4 embryos (i.e. 40 embryos in total) See detected region in D. (C) Mean computation time per image (error bars show standard deviation) for the different conditions using the same hardware as in the main text. The computation time for each image scales with the number of templates. (D) Montage of the detected regions for 10 images similar to A, each containing 4 embryos (1 column/image). The montage corresponds to the benchmark “1 Template + transformations” as in B and C. Yellow bounding boxes indicate the 2 detections classified as Partial in B. [file 12859_2020_3363_MOESM10_ESM.pptx]

## Slide 1
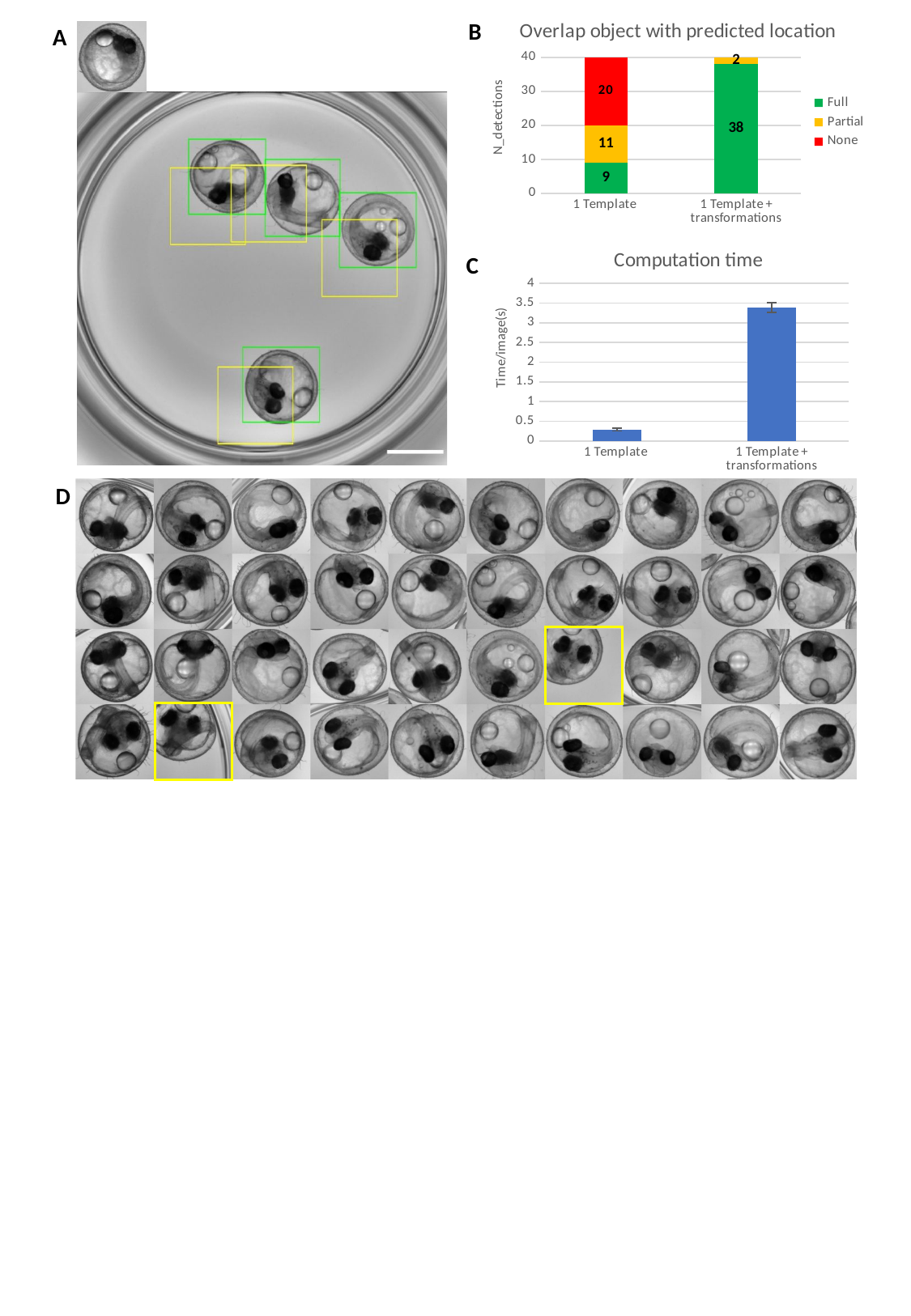

B
A
### Chart: Overlap object with predicted location
| Category | Full | Partial | None |
|---|---|---|---|
| 1 Template | 9.0 | 11.0 | 20.0 |
| 1 Template + transformations | 38.0 | 2.0 | 0.0 |
### Chart: Computation time
| Category | |
|---|---|
| 1 Template | 0.2885000000000001 |
| 1 Template + transformations | 3.389583333333333 |C
D
