# Supplementary material for: Multi-template matching: a versatile tool for object-localization in microscopy images
Source: BMC Bioinformatics. 2020 Feb 5;21:44. doi: 10.1186/s12859-020-3363-7 (PMC7003318; doi:10.1186/s12859-020-3363-7)
Supplement: Supplementary file 11 — Additional file 11: Figure S8. Multi-template matching for simultaneous head and trunk region detection in oriented zebrafish larvae. (A) Head (188 × 194 pixels) and trunk region (264 × 192 pixels) templates. Image (2048 × 2048 pixels, scale bar: 1 mm) in which the search is performed. The orange rectangle shows the optionally used restricted search region (1820 × 452 pixels). Parameters for the detection: Vertical flipping of the templates - score type: 0-mean normalised cross-correlation – N = 2 expected objects per image – score threshold: 0.6 – maximal overlap between bounding boxes: 0.35. (B) Result of the detection for N = 96 images, with and without search region. (C) Montage of the detected head regions in 96 zebrafish larvae when the search region is used. The head region was not detected in 3 specimens, 2 of them were not properly dorsally aligned. (see. Additional file 7: Figure S4C). (D) Montage of the detected trunk regions in 96 zebrafish larvae when the search region is used. When 2 trunks were detected in one image (instead of one trunk and one head), the trunk with the best score was used for the montage. Prior information about the position of the sample within the field of view (e.g. due to standardized sample mounting) can be used to specify a search region, drastically accelerating the computation and reducing the chance of incorrect predictions. (E) Mean computation time per image (N = 96 - error bars show standard deviation) for the different conditions as in B using the same computing hardware as in the main text. [file 12859_2020_3363_MOESM11_ESM.pptx]

## Slide 1
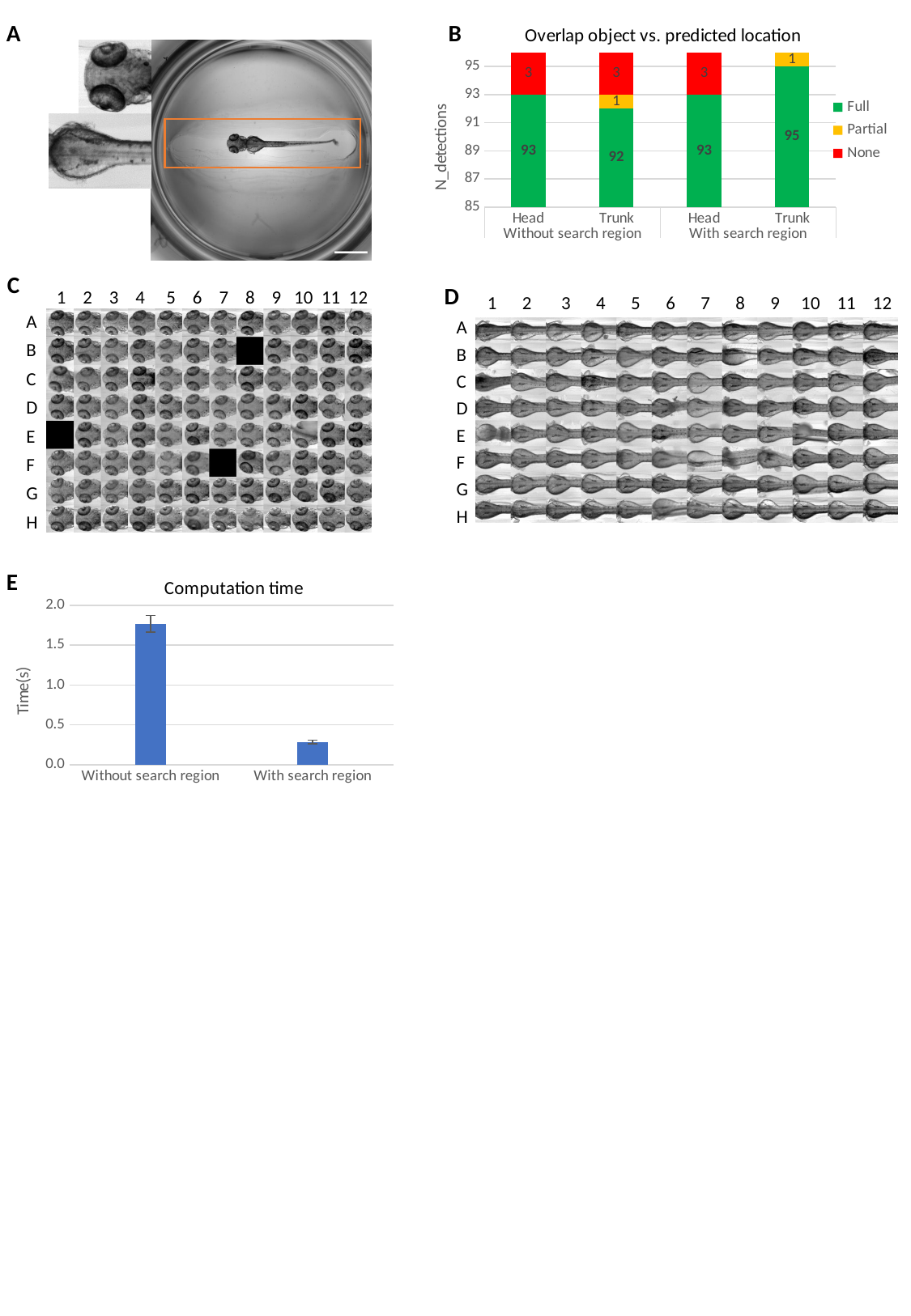

### Chart: Overlap object vs. predicted location
| Category | Full | Partial | None |
|---|---|---|---|
| Head | 93.0 | 0.0 | 3.0 |
| Trunk | 92.0 | 1.0 | 3.0 |
| Head | 93.0 | 0.0 | 3.0 |
| Trunk | 95.0 | 1.0 | 0.0 |A
B
C
1 2 3 4 5 6 7 8 9 10 11 12
1 2 3 4 5 6 7 8 9 10 11 12
D
A
B
C
D
E
F
G
H
A
B
C
D
E
F
G
H
E
### Chart: Computation time
| Category | Average |
|---|---|
| Without search region | 1.76896875 |
| With search region | 0.28537500000000005 |
